# Distinction of salvaged and infarcted myocardium within the ischemic area at risk with T2 mapping

**DOI:** 10.1186/1532-429X-16-S1-M3

**Published:** 2014-01-16

**Authors:** Sophia Hammer-Hansen, Martin Ugander, Li-Yueh Hsu, Joni Taylor, Peter Kellman, Andrew E Arai

**Affiliations:** 1 National Institutes of Health, Bethesda, Maryland, USA; 2 Karolinska Institute and Karolinska University Hospital, Stockholm, Sweden

## Background

Area at risk measurements often rely on T2 weighted images, but subtle differences in T2 may be overlooked with this method. Quantitative T2 mapping may bring us beyond some of the technical limitations associated with T2-weighted images (Giri et al JCMR, 2009). We hypothesize that T2 quantification can detect differences between salvaged and infarcted myocardium within the AAR in a reperfused model of acute myocardial infarction.

## Methods

Dogs underwent 2 hours of coronary occlusion followed by 4 or 48 hours of reperfusion before imaging. CMR imaging was performed at 1.5T (Siemens) using native T2 mapping, T2-prepared SSFP imaging, and late gadolinium enhancement (LGE). One midventricular slice was chosen per animal for analysis. LGE images were used to define infarcted, salvaged, and remote myocardial ROIs. Another ROI of both the infarcted and salvaged areas was defined as the AAR. Data were analyzed with ANOVA and Bonferroni correction for multiple testing. A p value < 0.05 was considered significant.

## Results

**Figure 1:**
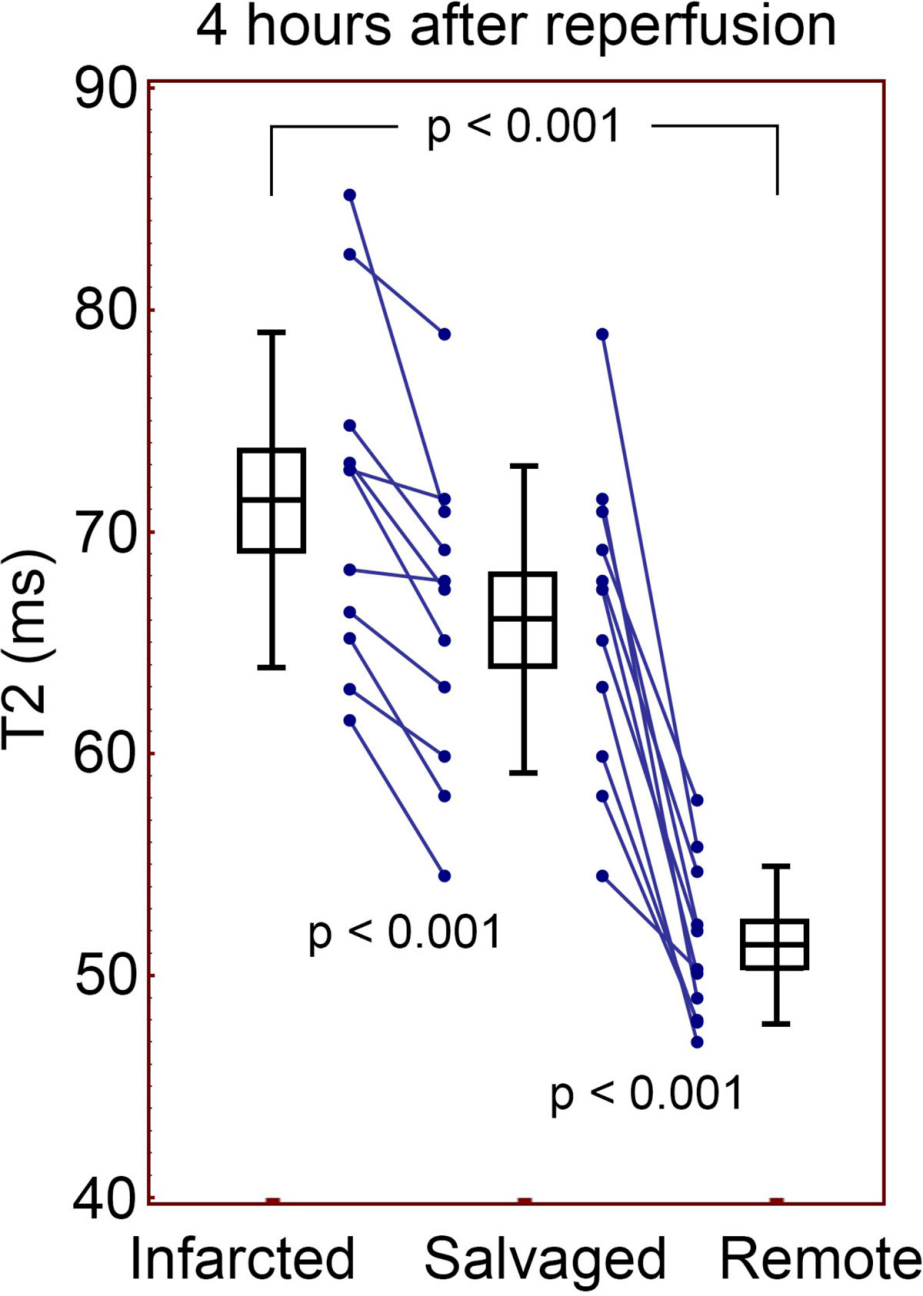
**T2 mapping was able to distinguish infarcted, salvaged, and remote myocardium 4 hours after reperfusion**. Boxes indicate mean and SEM, error bars indicate SD.

**Figure 2:**
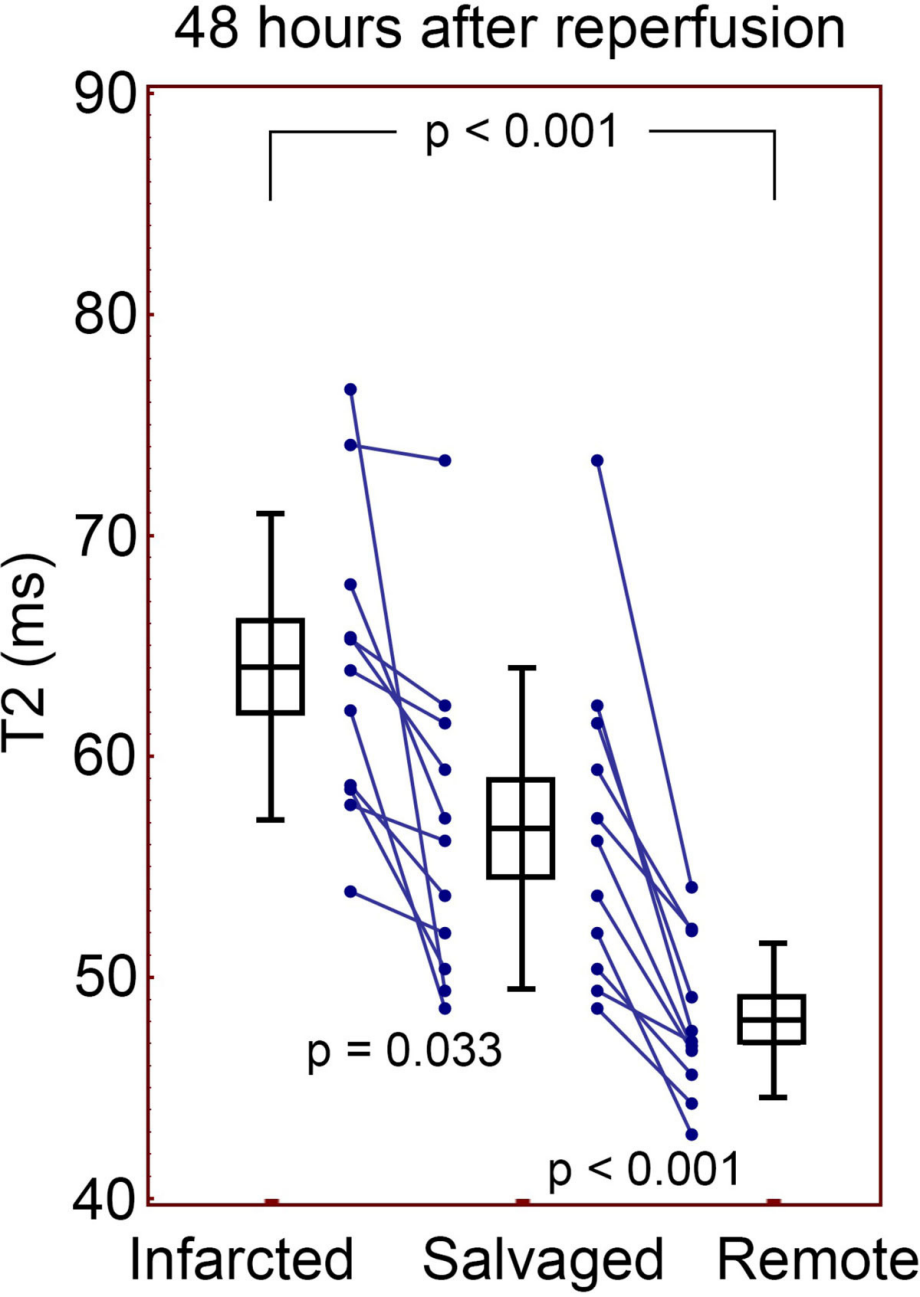
**T2 quantification after 48 hours of reperfusion**. Still the salvaged and infarcted myocardium are significantly higher than remote. Boxes indicate mean and SEM, error bars indicate SD.

22 animals were imaged after 4 (n = 11) or 48 hours (n = 11) of reperfusion. The signal intensity of the AAR was significantly greater than the remote myocardium on T2-prepared SSFP images, both at 4 hours (366 ± 57 vs. 253 ± 35, p < 0.0001) of reperfusion and 48 hours (572 ± 136 vs. 436 ± 114, p = 0.001). This was also the case with T2 quantification of the AAR compared to remote at 4 hours (69.3 ± 7.1 ms vs. 51.4 ± 3.5 ms, p < 0.0001)and 48 hours (60.1 ± 6.0 ms vs. 48.1 ± 3.5 ms, p < 0.0001). Dividing the AAR into infarcted and salvaged myocardium demonstrated that the T2 of salvaged myocardium was significantly longer than remote myocardium at both 4 and 48 hours of reperfusion. The T2 of infarcted myocardium was also longer than remote myocardium (see Figures 1 and 2). This was consistent with signal intensity data from the T2-weighted images (data not shown). A significant difference between the salvage and infarct was detectable at both time points as well (see figures). Interestingly, the ΔT2salvage(T2salvage-T2remote) was greater after 4 hours than after 48 (14.7 ± 5.6 ms vs. 8.7 ± 5.1 ms, p = 0.016), respectively.

## Conclusions

T2 mapping techniques quantitatively differentiated sub-regions within the AAR during the first days of reperfusion. The T2 of salvaged myocardium was significantly higher than remote myocardium after both 4 and 48 hours of reperfusion, though the magnitude of the difference was greater at 4 hours. T2 mapping was also able to distinguish salvaged from infarcted myocardium.

## Funding

Funded by the Intramural Research Program of the National Heart, Lung, and Blood Institute of The National Institutes of Health.

